# Research progress of Nedd4L in cardiovascular diseases

**DOI:** 10.1038/s41420-022-01017-1

**Published:** 2022-04-16

**Authors:** Mohan Li, Guozhe Sun, Pengbo Wang, Wenbin Wang, Kexin Cao, Chunyu Song, Yingxian Sun, Ying Zhang, Naijin Zhang

**Affiliations:** 1 Department of Cardiology, First Hospital of China Medical University, Shenyang, Liaoning China; 2 Institute of Health Sciences, China Medical University, Shenyang, Liaoning China

**Keywords:** Ubiquitylation, Ubiquitylation

## Abstract

Post-translational modifications (PTMs) are a covalent processing process of proteins after translation. Proteins are capable of playing their roles only after being modified, so as to maintain the normal physiological function of cells. As a key modification of protein post-translational modification, ubiquitination is an essential element, which forms an enzyme-linked reaction through ubiquitin-activating enzyme, ubiquitin binding enzyme, and ubiquitin ligase, aiming to regulate the expression level and function of cellular proteins. Nedd4 family is the largest group of ubiquitin ligases, including 9 members, such as Nedd4-1, Nedd4L (Nedd4-2), WWP1, WWP2, ITCH, etc. They could bind to substrate proteins through their WW domain and play a dominant role in the ubiquitination process, and then participate in various pathophysiological processes of cardiovascular diseases (such as hypertension, myocardial hypertrophy, heart failure, etc.). At present, the role of Nedd4L in the cardiovascular field is not fully understood. This review aims to summarize the progress and mechanism of Nedd4L in cardiovascular diseases, and provide potential perspective for the clinical treatment or prevention of related cardiovascular diseases by targeting Nedd4L.

## Facts

- Nedd4L, containing a C2 domain, four WW domains, and a HECT domain, is involved in various pathophysiological processes, and exert distinct influences on various cardiovascular diseases including hypertension, dilated cardiomyopathy, diabetic cardiomyopathy, myocardial infarction, heart failure, etc.
- Nedd4L is a key regulator of epithelial sodium channel (ENaC) activity.
- Targeting Nedd4L might be the hopeful option for target organ protection in patients with hypertension in the future.
- Mutations of the sodium voltage-gated channel alpha-subunit 5 (SCN5A) gene encoding the NaV1.5 protein leads to the occurrence of cardiomyopathy and arrhythmia.

## Introduction

Post-translational modifications (PTMs) of proteins are the covalent addition of functional groups or proteins. The proteolytic cleavage of regulatory subunits, or the degradation of the entire protein could ultimately change the structure, conformation, and physicochemical properties of proteins [1]. This process could further facilitate the functional diversity of modified proteins from the genome level to the proteome. These modifications mainly included phosphorylation [2], acetylation [3], and ubiquitination [4–6]. As we know, the addition of ubiquitin to substrate proteins is called ubiquitination. Ubiquitin [7] is a low molecular weight protein with highly conserved evolution. Yeast and human ubiquitin only differs in three amino acids [8, 9]. Ubiquitination can mark the target proteins, allow them to be recognized and degraded by the proteasome. Hence, as an important and common form of protein post-translational modification [10, 11], ubiquitination modifications usually occur on lysine residues and thereby regulate the various cellular pathways. Previous studies have mostly focused on upstream proteins such as transcription factors, but the regulatory mechanisms of upstream proteins are complex and there are many factors affecting this process. Research works from our team focused on the most important downstream factors of cellular damage and found that protein ubiquitination modifications played important roles on target organ protection through modulating neuronal precursor cell expression developmentally downregulated 4 (Nedd4) family members [12–14]. The whole process of ubiquitination is formed by a ubiquitin-proteasome system(UPS), which is mainly composed of ubiquitin-activating enzyme (E1), ubiquitin cross-linking enzyme (E2), ubiquitin ligase (E3) and 26S proteasome [15]. The NEDD4 family is one of the most essential groups of E3 ligase [9, 16], which includes neuronal precursor cell expression developmentally downregulated 4-1 (RPF1), neuronal precursor cell expression developmentally downregulated 4-like (Nedd4L/Nedd4-2), ITCH/atropine-1 interacting protein 4 (AIP4) and etc [17–19]. The ubiquitination process is formed by the enzyme-linked reaction consisting of the following three ubiquitinase [20]: 1. In the case of energy supplying by ATP, ubiquitin-activating enzyme E1 could activate ubiquitin molecules; 2. Ubiquitin-activating enzyme E1 then transfers the activated ubiquitin molecules to ubiquitin-conjugating enzyme E2 ; 3. Ubiquitin ligase E3 finally connects the E2-binding ubiquitin to the target protein [21, 22]. Being the most important enzyme in the ubiquitination process, E3 ligase is presented in three families: RING (really interesting new gene) family [23], E3 enzymes with HECT domain (homologous to E6-associated protein C-terminus E3 ligase, HECT E3S) [24–26], and the U-box family [27]. HECT E3S can be divided into three subfamilies based on the presence of different amino acid sequence motifs or domains at the N-terminus [25]: (1) Neuronal precursor cell expression developmentally downregulated 4 (Nedd4) subfamily (9 members) [17, 28], containing tryptophan Acid-tryptophan Acid (WW) motif [29]; (2) HERC (HECT and RCC1-like domains) subfamily (6 members), possessing one or more chromosome condensation 1 (RCC1)-like domains (RLDs) [30, 31]; (3) "Other" HECTs (13 members), containing various domains as well [32, 33]. Namely, Nedd4L is a member of the Nedd4 subfamily of HECT E3S of the ubiquitin-proteasome system(UPS) [34]. Nedd4L is located to human chromosome 18q21(ref. [35]), and Nedd4L in human is homologous to mouse Nedd4-2(ref. [36, 37]). Nedd4L is widely expressed during mouse development [38] and is also involved in the pathogenesis of cardiovascular diseases through multiple pathways. It is composed of C2 domain [39], four WW domains [40, 41] and one HECT domain. During the last decades, the incidence of cardiovascular diseases increased dramatically worldwide [42, 43]. In the past few years, the association between Nedd4L and cardiovascular diseases has been focused by many investigators. Results showed that Nedd4L could regulate cardiac function through various pathways and modulate a series of related physiological and pathological processes. In this article, we summarized the recent progress on the role of Nedd4L in various cardiovascular diseases.

## Nedd4L and hypertension

### Interaction of Nedd4L and ENaC and the potential role on sodium homeostasis and blood pressure

Hypertension is a common disease in the cardiovascular field [44–47], and is an important risk factor for stroke, myocardial infarction, heart failure, and kidney damage. Therefore, the pathogenesis of hypertension is a long-standing research focus [48–51]. Accumulating evidence show that, Nedd4L is involved in the formation and development of hypertension at the molecular level. The association between sodium intake, sodium hemostasis in human body and hypertension is well known now. The presence of Nedd4L in the kidney indicates its role and the interaction on sodium reabsorption in the kidney, thus affecting the blood pressure [52, 53]. A previous study demonstrated that reabsorption of Na^+^ [54] could be regulated by the Nedd4 family member-- epithelial sodium channel (ENaC) in the kidney, an important regulator of electrolyte balance and fluid stability [55].

ENaC is structurally composed of three subunits (alpha-, beta-, and gamma-), each containing a conserved proline-tyrosine (PY) motif at the C-terminal. Nedd4 protein could bind to these PY motifs on ENaC, and undergo ubiquitination through its WW domain [56]. Activated ENaC is capable of mediating the transportation of Na^+^ through the epithelial cells in the kidney, intestine, and lung [38, 57, 58]. Meanwhile, some biochemical and overexpression experiments have shown that Nedd4L could bind to the β- and γ-subunits of ENaC through its PY motif in the C-terminal region, which could then interact with the WW domain of Nedd4L and undergo ubiquitination for degradation [59, 60]. Phosphorylation of Nedd4L by serum and glucocorticoid-regulated kinase 1 (SGK1), for instance, may also regulate its combination with ENaC, then ubiquitinating ENaC. Thus, the interaction between Nedd4L and ENaC might play an important role in sodium homeostasis, and the regulation of blood pressure (BP) [57]. Therefore, it is of importance to further define the role and mechanism of Nedd4L ubiquitination in the protection of target organs in hypertension. It remains unknown if targeting Nedd4L could be the hopeful option for target organ protection of hypertension.

### Nedd4L and essential hypertension

Essential hypertension (EH) is common, but the underlying pathogenesis is complex and still not fully understood. Genetic variants are also considered to be one of the causes of EH. In addition, epigenetic, environment and other factors might also be responsible for the pathogenesis of EH [61]. Among them, post-translational histone modifications are considered to be the crucial epigenetic marks associated with EH [62]. At present, more and more members of the Nedd4 family in the ubiquitinated proteasome system, especially Nedd4L, are constantly being explored for their role in EH through both experimental and clinical studies [38, 63, 64]. It is shown that the Nedd4L gene may be a candidate gene that causes high blood pressure in humans. Nedd4L has three isoforms (isoform I, II, and III). Studies found that the interaction of isoform I might interact with other human isoforms and abnormally increase sodium reabsorption [38]. Loffing-Cueni et.al. have found that Nedd4L is highly expressed in aldosterone-sensitive distal nephron (ASDN) near to the collecting duct [65], indicating its role on hypertension. A previous study in African Americans found that the A allele of SNP rs4149601 was associated with increased blood pressure, with the involvement of Nedd4L. The detailed mechanism may be that this allele could reduce the ubiquitination and degradation of epithelial Na^+^ channels, resulting in an increase in the density of epithelial sodium channels or prolonged residence time on the cell surface, which will further lead to the increasing of the epithelial sodium transportation, eventually result in hypertension. Russo et.al. also found two Nedd4L SNPs (rss513563 and rs3865418) were associated with hypertension in American whites, and two other Nedd4L SNPs (rs4149589 and rs3865418) were associated with hypertension in Greek whites [66]. Three representative Nedd4L variants (296921-296923delTTG, rs2288774, and rs2288775) were found to be related to EH in the general Kazakh population in a case survey study among Kazakh women, suggesting that the Nedd4L gene variant may be associated with the essential hypertension in Kazakh’s women [61].

### Nedd4L and salt-sensitive hypertension

**Fig. 1:**
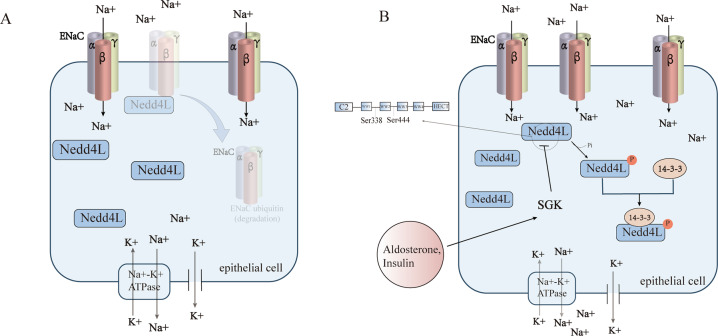
The mechanism diagrams of how SGK1 regulates sodium reabsorption by Nedd4L. **A** Epithelial sodium channel (ENaC) can be degraded by Neuronal precursor cell expression developmentally downregulated 4-like (Nedd4L) to maintain sodium balance. **B** Serum and glucocorticoid-regulated kinase 1(SGK1) phosphorylates and inhibits Nedd4L which is an ubiquitin ligase, thereby failing to reduce the channel expression and stimulate ion channel degradation. The phosphorylation (mainly surrounding amino acids Ser444, Ser338) mediated by SGK1 induces the interaction of Nedd4L with members of the 14-3-3 protein family, which in turn disrupts the ubiquitination and degradation of ENaC by Nedd4L, ultimately leading to more sodium reabsorption.

The so-called salt-sensitive hypertension refers to increased blood pressure by relatively high-salt intake [47, 67]. Studies have shown that SGK1 may play a role in the pathogenesis of salt-sensitive hypertension, SGK1 phosphorylates and inhibits Nedd4L which is an ubiquitin ligase, thereby failing to reduce the channel expression and stimulate ion channel degradation. The phosphorylation mediated by SGK1 induces the interaction of Nedd4L with members of the 14-3-3 protein family, which in turn disrupts the ubiquitination and degradation of ENaC by Nedd4L [68, 69] (Fig. 1**)**. It can be said that SGK1, functioning as a mediator, indirectly activates ENaC in tubules [70]. In salt-sensitive hypertensive rats, ENaC is abnormally regulated by aldosterone [71], contributing to the initiation and progression of salt-sensitive hypertension [72, 73]. In recent years, accumulating evidence defined the interaction between NEDD4L and ENaC and their role in the regulation of blood pressure. Nedd4L is now considered to be an E3 ubiquitin ligase involved in the process of salt-sensitive hypertension [74]. It is involved in the control of the sodium transporters, which are distinct from the epithelial sodium channels, but also play a determinant role in salt-sensitive hypertension [75]. A previous study showed that conditional Nedd4L knockout mice showed similar blood pressure as control mice under standard diet, but blood pressure was significantly higher in conditional Nedd4L knockout mice than in control mice after 12 days high-Na^+^ diet, accompanied by higher urinary calcium. [71] Another study evidenced higher blood pressure in Nedd4L knockout mice even under normal diet, and blood pressure was further increased under a high-salt diet, studies also found that salt-sensitive hypertension in the Nedd4L-C2 (KO) mice was resistant to eplerenone (EPL) [57]. Aldosterone-independent transcriptional activation of ENaC in the aldosterone-sensitive distal nephron (ASDN) was evidenced in Nedd4L-C2 KO mice, this activation could be abolished by Amiloride [76]. The following factor might be involved in the induction of hypertension in Nedd4L knockout mice: Upregulated expression levels of all three ENaC subunits in the kidneys [57]. However, a specific inhibitor of sodium ion channel, amiloride, can reverse the elevated blood pressure shown in the Nedd4L-KO mice under high-salt diet [76]. The above results indicate that Nedd4L is a key regulator of ENaC activity and blood pressure in vivo. In fact, studies have found that carriers of the Nedd4L salt-sensitivity-associated genotype (rs4149601 A→G and rs2288774 T→C NEDD4L variants) had higher systolic and diastolic blood pressure and multivariate adjusted hazards ratio (95% confidence interval) of CVD 1.13 (1.02-1.25, *P* = 0.018), coronary events 1.20 (1.06-1.37; *P* = 0.005) and cardiovascular mortality 1.17 (0.99-1.37; *P* = 0.055) than noncarriers, suggesting that the genotype of Nedd4L related to salt-sensitivity is associated with hypertension and higher risk of cardiovascular morbidity and mortality in human beings [77]. Nedd4L was once considered as a candidate protein to control the surface expression of ENaC, but which component is indeed involved in the regulation of ENaC in epithelial cells was unknown. Through RNA interference technology, Snyder and colleagues discovered that endogenous Nedd4L could negatively regulate ENaC in epithelial cells, which is the key component of steroid hormone-regulated ENaC signaling pathway, so the defect of this regulation pathway may be directly linked with the pathogenesis of hypertension [78].

### Nedd4L and Liddle syndrome

**Fig. 2:**
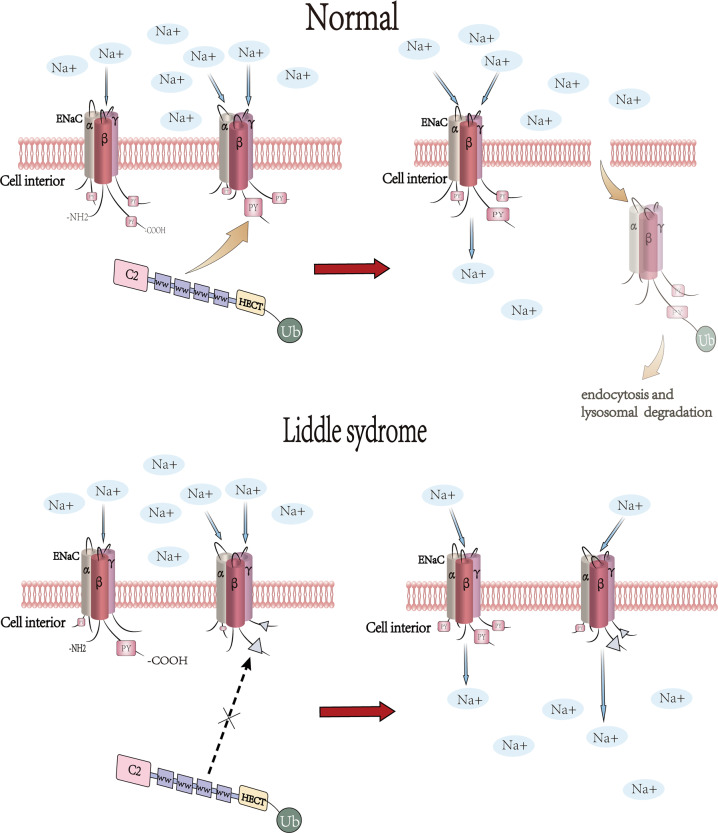
The molecular mechanism of the differences between the normal and liddle syndrome. Neuronal precursor cell expression developmentally downregulated 4-like(Nedd4L) is composed of C2 domain, four WW domains and HECT domain. α, β, and γ subunits of ENaC contains a conserved proline-tyrosine (PY) motif at the C-terminus which is also the binding site for the ubiquitin ligase Nedd4L. After mutation in this PY motif happens, the binding capacity to the WW domain of Nedd4L will decrease; thereby accelerate the cell activity and expression of ENaC, leading to its uncapable of degradation. Ultimately, increase the absorption of sodium ion in the distal nephron.

Liddle syndrome is characterized by hypertension with a background of hypokalemia and metabolic alkalosis [79, 80]. It is usually caused by abnormally elevated sodium reabsorption in distal nephrons. The α, β, and γ subunits of ENaC are known to contain a conserved proline-tyrosine (PY) motif at the C-terminus [56]. Mutation in this PY motif, which is also the binding site for the ubiquitin ligase Nedd4L, might cause Liddle syndrome [81, 82] (Fig. 2). In case of β or γ subunits deficiency of the PY motif, binding capacity to the WW domain of Nedd4L will decrease, thereby accelerate the cell activity of ENaC, promote apoptosis, increase the absorption of sodium ion and fluid in the distal nephron, and ultimately lead to the elevated blood volume and blood pressure [81, 83], implying that Nedd4L may be an inhibitor of epithelial Na^+^ channels [84]. Liddle syndrome can be manifested as severe hypertension, hypokalemia, metabolic alkalosis, and hyporeninemia clinically. It is an inherited form of salt-sensitive hypertension [56, 84, 85]. Capillary sodium channels located on the aldosterone-sensitive distal nephron (ASDN) also play a key role in the homeostasis of sodium homeostasis. Clinical studies have confirmed that Liddle syndrome is related to the gene mutation of ENaC-NEDD4L-proteasome. A clinical study showed that Nedd4L gene single nucleic acid polymorphisms (SNPs) were closely related to hypertension [35]. Abriel and colleagues evidenced the loss of the Nedd4 binding site in ENaC in Liddle syndrome through immunofluorescence and other methods [86]. In addition, knockout of Nedd4L protein from adult mouse renal tubules can lead to the accumulation of ENaC, which may lead to increased cell surface channels and sodium reabsorption in distal nephrons, and ultimately resulting in hypertension [86]. The human Nedd4L gene, especially the evolutionary new subtype I, is a candidate gene for hypertension [38]. Certain small-molecule compounds could destabilize cell-surface ENaC or enhance the Nedd4L activity in the kidney, thus being hopeful candidates for anti-hypertension agents [87].

### Nedd4L and myocardial remodeling

#### Nedd4L and cardiac diseases

Cardiac hypertrophy and dilated cardiomyopathy, as a type of myocardial remodeling [88–90], is one of the risk factors for heart failure (HF) [91–93]. Up till now, heart failure has been the main leading cause of death in the cardiovascular field [94, 95]. Nedd4L is the key player not only responsible for the regulation of blood pressure but also for the electrolyte balance in vivo. Cardiac hypertrophy and significantly decreased cardiac function were detected in Nedd4L knockout mice fed with chronic high-salt diet [57]. It is known that the density and expression of sodium channels in vivo play an important role in the pathogenesis of cardiovascular diseases. Among the ion channels of the heart, sodium voltage-gated channel alpha-subunit 5 (SCN5A) encodes the Na_V_1.5 protein [96, 97]. As an essential transmembrane channel protein, Na_V_1.5 is structurally composed of 4 similar domains, each of which contains 6 transmembrane proteins (S1-S6) linked by amino acid sequences [98]. The exact cause of dilated cardiomyopathy cannot always be found clinically, approximately 50 variants have been identified in patients with DCM that cause macroscopically dilated cardiomyopathy. This seems to imply that the occurrence of DCM is closely related to genetic background [99]. In 2008, Ge and his team conducted a research on a Chinese DCM pedigree and found that the cardiac sodium channel gene (SCN5A) A1180V was associated with the disease development in the whole family [97]. In basic researches, some scholars found that the mutation of SCN5A related to the occurrence of DCM is mainly distributed in the DI-S4 segment of the Nav1.5 protein, while the A1180V mutation occurs at DII-DIII segment [99, 100]. In fact, the electrophysiological damage associated with DCM is now thought to be caused by its variant which reduces peak sodium currents [99]. In addition, A1180V induces a 4.5mV negative inactivation offset of the mutant channel and also shows a slower recovery. This emergence of cardiac sodium ion current disturbance can greatly aggravate myocardial injury and ultimately lead to dilated cardiomyopathy, which in turn leads to heart failure [97]. Ge and his team also found that participants carrying the A1180V gene face higher risk for disease progression, and the mechanism might relate to changed sodium ion balance, resulting in electronic disorders and ultimately leading to structural heart disease [97, 101].

It is known that endocrine-related diseases such as diabetes [102–104] and insulin resistance [105] can also cause diabetic cardiomyopathy [106, 107]. The role of Nedd4L in the development of diabetic cardiomyopathy has also been explored in recent years. Nedd4L can become the phosphorylated form of Nedd4L, this process is mediated by SGK1. It acts as a direct downstream target of SGK1, and down-regulating SGK1 levels could downregulate Nedd4L and vice versa. Shi et al. found that high glucose may downregulate the expression of SGK1, resulting in the reduction in the phosphorylated form of Nedd4L (an inactive state). This implies that SGK1 and Nedd4L are involved in the development of hyperglycemia-induced cardiomyopathy, future studies are warranted to validate whether targeting SGK1 and Nedd4L could provide a solution for the clinical treatment of diabetic cardiomyopathy or not [108].

#### Nedd4L and myocardial infarction

Myocardial infarction (MI) is related to the highest mortality rate among cardiovascular diseases [109–111]. Myocardial ischemia caused by MI, and heart remodeling [112] could be the causes of clinical death. Studies have shown that the loss of Nedd4L-C2 subtype can cause the cardiac conduction changes in resting state and responsible for the pro-arrhythmic change after acute myocardial infarction [113]. From a prognostic point of view, Huang and his colleagues found that the downregulation of circular RNA Nfix (circNfix) in cardiomyocytes can attenuate the process of myocardial remodeling after MI and partially restore myocardial functions. Y-box-binding protein 1 (Ybx1) [114] is an essential transcriptional protein in myocardial proliferation after myocardial infarction, which acts as a substrate for Nedd4L and can bind to Nedd4L for degradation. Studies have found that circNfix could bind Nedd4L. When circNfix is overexpressed, the binding of Nedd4L and circNfix can be promoted, resulting in the reduction of Ybx1. Downregulation of circNfix could upregulate Ybx1, promote proliferation and regeneration of cardiomyocytes, ultimately improve prognosis post-MI [115]. The in-depth understanding of the above mechanisms provides a theoretical basis for the clinical targeted therapy of patients with MI in the future, and also provides inspiration for new treatment approaches.

Heart failure which is a common heterogeneous clinical syndrome. Ubiquitination also played an important role in heart failure. Luo et.al. found that microenvironment change could lead to degradation changes of ENac by Nedd4L. When the intracellular concentration of calcium ion increases, the activity of Nedd4L increases, thereby promoting the degradation of the sodium ion channels located on the membrane. In addition, Nedd4L and its colocalization with sodium channels are also increased in case of heart failure [116]. It is also found that after the occurrence of HF, the expression of Nav1.5 protein was significantly downregulated [117]. A previous study demonstrated that miR-454 could affect heart failure (HF) progression by modulating Nedd4L/tropomyosin receptor kinase A (TrkA)/cyclic adenosine 3′,5′-monophosphate (cAMP) axis, in that miR-454 exerts anti-apoptotic and protective effects on cardiomyocytes by inhibiting Nedd4L, while Nedd4L stimulates the ubiquitination and degradation of TrkA protein. The miR-454 activates the cAMP pathway through the Nedd4L/TrkA axis, which ultimately inhibits cardiomyocyte apoptosis and alleviates myocardial injury. Results from this landmark study thus suggest that targeting the Nedd4L/TrkA/cAMP pathway may be a potential novel promising option for the prevention and treatment of heart failure [118].

#### Nedd4L and arrhythmias

The sodium channel has three subunits, α, β, and γ [56]. Sodium voltage-gated channel alpha-subunit 5 (SCN5A) (Table 1) encodes the voltage-dependent alpha-subunit of NaV1.5 protein [97, 98]. Nedd4L binds through its WW domain to the PY motif at the C-terminus of Na_V_1.5 protein encoded by SCN5A [59, 119].This results in decreased protein expression and current through Na_V_1.5 ubiquitination [113, 120]. The above can indicate that the mutation of the SCN5A gene encoding Na_v_1.5 protein or the changes of its expression level are closely related to the occurrence of diseases with abnormal electrophysiological conduction under various factors [121]. Therefore, it is of importance to explore the mechanism of Na_V_1.5 channel mutation for the prevention and treatment of arrhythmia, especially malignant arrhythmias [120, 122, 123].

**Table 1:** This table shows how SCN5A, which encodes the NaV1.5 channel, plays a role in cardiomyopathies and arrhythmias (only includes well-studied diseases), most of which are associated with variants in SCN5A.

Mechanisms of the variant	Biological responses	References
**The role of SCN5A gene encoding Nav1.5 protein in cardiovascular diseases (Only mention diseases for which there is a definite finding)**		
*Cardiomyopathy*		
SCN5A-A1180V mutation, occurs at DII-DIII segment, induces a 4.5mV negative inactivation offset of the mutant channel and also shows a slower recovery.	Dilated cardiomyopathy (DCM)	[97, 99, 100]
*Arrhythmia*		
SCN5A variants may lead to the decreasing of cardiac excitability, resulting in the loss-of-function property of diminished electrical coupling between the sinoatrial node and surrounding atrial cells.	sick sinus syndrome (SSS)	[96, 124, 125]
The eight novel variants of SCN5A, including M138I, F428K, H445D, N470K, E655K, T113I, R1826C, V1951M, were all associated with the occurrence and susceptibility of AF.	Atrial fibrillation (AF)	[127, 128]
The decreased expression of atrial-specific gap junction protein connexin40 (Cx40); the mutations in the extremely important sodium ion channel gene SCN5A D1275N in the heart.	Atrial standstill (AS)	[129]
Brs is a heritable channelopathy. The SCN5A variants associated with BrS are usually located in the region between DI and DII. More than 300 SCN5A variants have been found to be associated with this inherited disease.	Brugada syndrome (Brs)	[133, 134]
SCN5A-p.Y1977N disrupts the common Nedd4L binding site (from PPxY to PPxN), thus prevent the process of ubiquitination.	Long QT syndrome	[135, 136]

In the field of the arrhythmia associated with the atrium, studies have found that mutations in SCN5A are associated with diseases such as sick sinus syndrome (SSS) [124]. Loss or enhancement of sodium channel function caused by SCN5A variants can lead to the decreasing of cardiac excitability, resulting in sinus node dysfunction. The most typical state caused by SCN5A variability is the loss of function of the sodium ion channel. In the molecular level, it is manifested as the loss-of-function property of diminished electrical coupling between the sinoatrial node and surrounding atrial cells, resulting in outlet blocking, which is a common character in SSS [96, 125]. The other diseases most associated with SCN5A are currently found to be atrial fibrillation (AF) and atrial standstill. AF has always been the most common type of cardiovascular system disease in arrhythmia [126]. Clinically, its electrocardiogram often shows rapid irregular atrial fibrillation waves of 350–600 beats per minute. Experts have found that among patients diagnosed with AF, those with a family history of atrial fibrillation are more likely to develop new-onset AF [127], which implies that the occurrence of AF may be closely related to genetic factors [128]. In addition, studies have found that mutations or rare variants in the SCN5A gene may predispose people without underlying heart disease to AF. Hence, it seems that the susceptibility of AF is related to the variations in the gene named SCN5A which encoding the cardiac sodium channels [127]. However, AS is a rare arrhythmia [129]. In 2003, Groenewegen et al. investigated families with this rare disease background and found that the occurrence of AS was related to the decreased expression of atrial-specific gap junction protein connexin40 (Cx40), and the extremely important sodium ion channel gene SCN5A D1275N in the heart also has mutations. Hence the emergence of AS is also considered to appear under the joint influence of various factors [129].

Secondly, in the direction of arrhythmia related to the ventricle, there are also many arrhythmias related to the mutation of the sodium channel encoding gene SCN5A. Among the primary arrhythmic syndromes (including Brugada syndrome, long QT syndrome, short QT syndrome, early repolarization syndrome, etc.) [130], Brugada syndrome (BrS) was found to be most associated with SCN5A [131]. In 1998, the variant of this gene encoding the sodium channel was discovered for the first time [132]. Eight allele-related inherited heart diseases have been found to be associated with the genetic mutations of SCN5A [133]. It has been found that the SCN5A variants associated with BrS are usually located in the region between DI and DII [134]. Currently, 21% of BrS probands are found to be carriers of SCN5A variants, and more than 300 SCN5A variants have been found to be associated with this inherited disease [134]. Other studies have found that the SCN5A-p.L1239P variant can enhance the ubiquitin and degradation of Na_V_ 1.5 channels due to the existence of a new binding site. Another variant, SCN5A-p.Y1977N, which disrupts the common Nedd4L binding site (from PPxY to PPxN), could prevent the process of ubiquitination, and this variant is associated with long QT syndrome [135, 136] (Table 2).

**Table 2:** This table shows the roles and functions of Nedd4L in various cardiac diseases, especially hypertension.

**Disease category**	**Involvement in the diseases**	**Functions and mechanisms**	**References**
***Roles and functions of Nedd4L in cardiac diseases***			
Hypertension	Salt-sensitive hypertension	SGKI phosphorylates and inhibits Nedd4L which is a ubiquitin ligase, thereby failing to reduce the channel expression and stimulate ion channel degradation. The phosphorylation mediated by SGK1 induces the interaction of Nedd4L with members of the 14-3-3 protein family, which in turn disrupts the ubiquitination and degradation of ENaC by Nedd4L, ultimately leads to salt-sensitive hypertension.	[68, 69]
	Essential hypertension	Nedd4L isoform I can interact with other isoforms and can increase sodium reabsorption, which can lead to hypertension; the A allele of SNP rs4149601 of Nedd4L was associated with increased blood pressure, with the involvement of Nedd4L. The detailed mechanism may be that this allele could reduce the ubiquitination and degradation of epithelial sodium channels, resulting in an increase in the density of epithelial sodium channels or prolonged residence time on the cell surface, which will further lead to the increasing of the epithelial sodium transportation, eventually result in hypertension.	[38, 66]
	Liddle syndrome	Mutations in the β or α subunits of the PY motif in the ENaC lead to the binding capacity to the WW domain of Nedd4L decreased, thereby accelerate the cell activity of ENaC, promote apoptosis, increase the absorption of sodium ion and fluid in the distal nephron, and ultimately cause the blood volume and blood pressure elevated.	[56, 81–83]
Cardiomyopathy	Dilated cardiomyopathy	SCN5A-A1180V induces a 4.5mV negative inactivation offset of the mutant channel and also shows a slower recovery. This emergence of cardiac sodium ion current disturbance can greatly aggravate myocardial injury and ultimately lead to dilated cardiomyopathy.	[97]
	Diabetic cardiomyopathy	The miR-195-5p/SGK1/Nedd4L axis plays an important regulatory role in high glucose-induced cardiomyopathy.	[108]
	Myocardial infarction	When the circNfix is overexpressed in cardiomyocytes, it can promote the binding of Nedd4L and Ybxl, resulting in the reduction of Ybxl, hence leads to the induction of cardiomyocyte proliferation, which is not beneficial to prognosis.	[114, 115]
	Heart failure	The miR-454 activates the cAMP pathway through the NEDD4L/TrkA axis, which ultimately inhibits cardiomyocyte apoptosis and alleviates myocardial injury.	[118]
Arrhythmia	Long QT syndrome	SCN5A-p.Y1977N disrupts the common Nedd4L binding site (from PPxY to PPxN), thus prevents the process of ubiquitination.	[135, 136]

(Studies imply that there is not much direct connection between Nedd4L and arrhythmia, and most of the arrhythmias are related to the gene SCN5A that encodes the sodium channel. Shown in Table 1) Here, eight diseases are listed in the table.

In addition, in a study of simulating myocardial infarction in Nedd4L-C2-KO mice, Minegishi et al. found that within 6 weeks after myocardial infarction in Nedd4L-C2-KO mice, the PR interval was significantly shortened compared with wild-type MI mice, QT interval and QTc was prolonged, T peak/T wave end interval showed an enhancement as well [113]. Experimental animal studies also showed that Nedd4L-C2-KO mice exhibited signs of bradycardia, QRS prolongation, QT interval prolongation, and suppressed PR interval in the resting state [113].

## Conclusion

In conclusion, Nedd4L, as the dominating E3 ligase of the Nedd4 family, and related signaling play fundamental roles in cardiovascular diseases. In this present review, we’ve already known that the ubiquitination ligase, Nedd4L, participates in various pathophysiological processes of cardiovascular diseases. The Nedd4L isoform is expressed in several organs, thus plays a key role in the post-transcriptional modification of sodium transporters and cardiac ion channels. The encoding gene SCN5A, ENaC, and the mediator SGK1 and etc. participate in the pathogenesis of various cardiac diseases. Either mutations or deletion of above genes could lead to the poor outcome. Several signaling pathways play important roles in regulating the body’s blood pressure, among them, NEDD4L is a key regulator of these processes. Therefore, understanding their roles and targeting these molecules might hint novel therapeutic approaches to various cardiovascular diseases.

## Supplementary information

Extended Data 1

## Data Availability

The [DATA TYPE] data used to support the findings of this study are available from the corresponding author upon request.

## References

[1] Basak S, Lu C, Basak A (2016). Post-translational protein modifications of rare and unconventional types: implications in functions and diseases. Curr Med Chem.

[2] Covian R, Balaban RS (2012). Cardiac mitochondrial matrix and respiratory complex protein phosphorylation. Am J Physiol Heart Circulatory Physiol.

[3] Issa Diallo MichelSeve, Valérie Cunin FrédéricMinassian, Jean-François Poisson SylvieMichelland (2019). Current trends in protein acetylation analysis. Expert Rev Proteom.

[4] Song L, Luo Z-Q (2019). Post-translational regulation of ubiquitin signaling. J Cell Biol.

[5] Zhang N, Zhang Y, Miao W, Shi C, Chen Z, Wu B (2022). An unexpected role for BAG3 in regulating PARP1 ubiquitination in oxidative stress-related endothelial damage. Redox Biol.

[6] Zhang N, Zhang Y, Wu B, You S, Sun. Y (2020). Role of WW domain E3 ubiquitin protein ligase 2 in modulating ubiquitination and Degradation of Septin4 in oxidative stress endothelial injury. Redox Biol.

[7] Yau R, Rape M (2016). The increasing complexity of the ubiquitin code. Nat Cell Biol.

[8] Goldstein G, Scheid M, Hammerling U, Schlesinger DH, Niall HD, Boyse EA (1975). Isolation of a polypeptide that has lymphocyte-differentiating properties and is probably represented universally in living cells. Proc Natl Acad Sci USA.

[9] Yang B, Kumar S (2010). Nedd4 and Nedd4-2: closely related ubiquitin-protein ligases with distinct physiological functions. Cell Death Differ.

[10] Powell, SR. The ubiquitin-proteasome system in cardiac physiology and pathology. Am J Physiol Heart Circulatory Physiol. 2006;291:H1–H19. 10.1152/ajpheart.00062.2006 16501026

[11] Shenoy. SK (2007). Seven-transmembrane receptors and ubiquitination. Circulation Res.

[12] Qian H, Zhang N, Wu B, Wu S, You S, Zhang Y (2020). The E3 ubiquitin ligase Smurf2 regulates PARP1 stability to alleviate oxidative stress-induced injury in human umbilical vein endothelial cells. J Cell Mol Med.

[13] Zhang N, Zhang Y, Qian H, Wu S, Cao L, Sun. Y (2020). Selective targeting of ubiquitination and degradation of PARP1 by E3 ubiquitin ligase WWP2 regulates isoproterenol-induced cardiac remodeling. Cell Death Differ.

[14] Wang P, Zhang N, Wu B, Wu S, Zhang Y, Sun. Y (2020). The role of mitochondria in vascular calcification. J Transl Int Med.

[15] Zhang X, Linder S, Bazzaro M. Drug development targeting the ubiquitin-proteasome system (UPS) for the treatment of human cancers. Cancers (Basel) 2020;12. 10.3390/cancers12040902. 10.3390/cancers12040902 PMC7226376 32272746

[16] Goel P, Manning JA, Kumar S. NEDD4-2 (NEDD4L): the ubiquitin ligase for multiple membrane proteins. Gene 2015;557. 10.1016/j.gene.2014.11.051. 10.1016/j.gene.2014.11.051 PMC6636357 25433090

[17] Anne Boase N, Kumar S (2015). NEDD4: The founding member of a family of ubiquitin-protein ligases. Gene.

[18] Scheffner M, Kumar S (2014). Mammalian HECT ubiquitin-protein ligases: biological and pathophysiological aspects. Biochim Biophys Acta.

[19] Zhang Y, Qian H, Wu B, You S, Wu S, Lu S (2020). E3 Ubiquitin ligase NEDD4 family‑regulatory network in cardiovascular disease. Int J Biol Sci.

[20] Zheng N, Shabek N (2017). Ubiquitin ligases: structure, function, and regulation. Annu Rev Biochem.

[21] Harvey KF, Kumar S (1999). Nedd4-like proteins: an emerging family of ubiquitin-protein ligases implicated in diverse cellular functions. Trends Cell Biol.

[22] Deshaies RJ, Joazeiro CAP (2009). RING domain E3 ubiquitin ligases. Annu Rev Biochem.

[23] Joazeiro CA, Weissman AM (2000). RING finger proteins: mediators of ubiquitin ligase activity. Cell.

[24] Rotin D, Kumar S (2009). Physiological functions of the HECT family of ubiquitin ligases. Nat Rev Mol Cell Biol.

[25] Goto J, Otaki Y, Watanabe T, Watanabe M. The role of HECT-Type E3 ligase in the development of cardiac disease. Int J Mol Sci 2021;22. 10.3390/ijms22116065. 10.3390/ijms22116065 PMC8199989 34199773

[26] Qian H, Zhang Y, Wu B, Wu S, You S, Zhang N (2020). Structure and function of HECT E3 ubiquitin ligases and their role in oxidative stress. J Transl Int Med.

[27] Paul Hutchins A, Liu S, Diez D, Miranda-Saavedra. D (2013). The repertoires of ubiquitinating and deubiquitinating enzymes in eukaryotic genomes. Mol Biol Evol.

[28] Woelk T, Oldrini B, Maspero E, Confalonieri S, Cavallaro E, Di Fiore PP (2006). Molecular mechanisms of coupled monoubiquitination. Nat Cell Biol.

[29] Shearwin-Whyatt L, Dalton HE, Foot N, Kumar. S (2006). Regulation of functional diversity within the Nedd4 family by accessory and adaptor proteins. Bioessays.

[30] Garcia-Gonzalo FR, Rosa JL (2005). The HERC proteins: functional and evolutionary insights. Cell Mol Life Sci.

[31] Sala-Gaston J, Martinez-Martinez A, Pedrazza L, Lorenzo-Martín LF, Caloto R, Bustelo XR, et al. HERC ubiquitin ligases in cancer. Cancers (Basel) 2020;12. 10.3390/cancers12061653. 10.3390/cancers12061653 PMC7352365 32580485

[32] Morreale FE & Walden H. Types of ubiquitin ligases. Cell 2016;165. 10.1016/j.cell.2016.03.003. 10.1016/j.cell.2016.03.003 27015313

[33] Singh S, Ng J, Sivaraman J (2021). Exploring the "Other" subfamily of HECT E3-ligases for therapeutic intervention. Pharm Ther.

[34] Varshavsky A (2017). The ubiquitin system, autophagy, and regulated protein degradation. Annu Rev Biochem.

[35] Russo CJ, Melista E, Cui J, DeStefano AL, Bakris GL, Manolis AJ (2005). Association of NEDD4L ubiquitin ligase with essential hypertension. Hypertension.

[36] Kamynina E, Debonneville C, Bens M, Vandewalle A, Staub O (2001). A novel mouse Nedd4 protein suppresses the activity of the epithelial Na+ channel. FASEB J.

[37] Chen H, Ross CA, Wang N, Huo Y, MacKinnon DF, Potash JB (2001). NEDD4L on human chromosome 18q21 has multiple forms of transcripts and is a homologue of the mouse Nedd4-2 gene. Eur J Hum Genet.

[38] Araki N, Umemura M, Miyagi Y, Yabana M, Miki Y, Tamura K (2008). Expression, transcription, and possible antagonistic interaction of the human Nedd4L gene variant: implications for essential. Hypertension Hypertension.

[39] Rizo J, Südhof TC (1998). C2-domains, structure and function of a universal Ca2+-binding domain. J Biol Chem.

[40] Ingham RJ, Colwill K, Howard C, Dettwiler S, Lim CSH, Yu J (2005). WW domains provide a platform for the assembly of multiprotein networks. Mol Cell Biol.

[41] Riling C, Kamadurai H, Kumar S, O’Leary CE, Wu K-P, Manion EE (2015). Itch WW domains inhibit Its E3 ubiquitin ligase activity by blocking E2-E3 ligase trans-thiolation. J Biol Chem.

[42] Andersson C, Vasan RS (2018). Epidemiology of cardiovascular disease in young individuals. Nat Rev Cardiol.

[43] Costantino S, Paneni F, Cosentino F (2016). Ageing, metabolism and cardiovascular disease. J Physiol.

[44] Coffman TM. Under pressure: the search for the essential mechanisms of hypertension. Nat Med. 2011;17:1402–9. 10.1038/nm.2541. 10.1038/nm.2541 22064430

[45] Hall JE, Granger JP, do Carmo JM, da Silva AA, Dubinion J, George E (2012). Hypertension: physiology and pathophysiology. Compr Physiol.

[46] Wang MC, Lloyd-Jones DM (2021). Cardiovascular risk assessment in hypertensive patients. Am J Hypertens.

[47] Mills KT, Stefanescu A, He J (2020). The global epidemiology of hypertension. Nat Rev Nephrol.

[48] Manosroi W, Williams GH (2019). Genetics of human primary hypertension: focus on hormonal mechanisms. Endocr Rev.

[49] He FJ, Tan M, Ma Y, MacGregor GA (2020). Salt reduction to prevent hypertension and cardiovascular disease: JACC state-of-the-art review. J Am Coll Cardiol.

[50] Colafella KMM, Denton KM (2018). Sex-specific differences in hypertension and associated cardiovascular disease. Nat Rev Nephrol.

[51] Safar ME. Arterial stiffness as a risk factor for clinical hypertension. Nat Rev Cardiol. 2018;15, 10.1038/nrcardio.2017.155. 10.1038/nrcardio.2017.155 29022570

[52] Farquhar WB, Edwards DG, Jurkovitz CT, Weintraub WS (2015). Dietary sodium and health: more than just blood pressure. J Am Coll Cardiol.

[53] Adrogué HJ, Madias NE (2007). Sodium and potassium in the pathogenesis of hypertension. N. Engl J Med.

[54] Snyder PM (2009). Down-regulating destruction: phosphorylation regulates the E3 ubiquitin ligase Nedd4-2. Sci Signal.

[55] Zhang D-D, Duan X-P, Xiao Y, Wu P, Gao Z-X, Wang W-H (2021). Deletion of renal Nedd4-2 abolishes the effect of high sodium intake (HS) on Kir4.1, ENaC, and NCC and causes hypokalemia during high HS. Am J Physiol Ren Physiol.

[56] Kamynina E, Debonneville C, Hirt RP, Staub O (2001). Liddle’s syndrome: a novel mouse Nedd4 isoform regulates the activity of the epithelial Na(+) channel. Kidney Int.

[57] Shi PP, Cao XR, Sweezer EM, Kinney TS, Williams NR, Husted RF (2008). Salt-sensitive hypertension and cardiac hypertrophy in mice deficient in the ubiquitin ligase Nedd4-2. Am J Physiol Ren Physiol.

[58] Dinudom A, Fotia AB, Lefkowitz RJ, Young JA, Kumar S (2004). The kinase Grk2 regulates Nedd4/Nedd4-2-dependent control of epithelial Na^+^ channels. Proc Natl Acad Sci USA.

[59] Hugues A (2007). Roles and regulation of the cardiac sodium channel Na v 1.5: recent insights from experimental studies. Cardiovascular Res.

[60] Kang Y, Guo J, Yang T, Li W, Zhang S (2015). Regulation of the human ether-a-go-go-related gene (hERG) potassium channel by Nedd4 family interacting proteins (Ndfips). Biochem J.

[61] Li N, Wang H, Yang J, Zhou L, Hong J, Guo Y (2009). Genetic variation of NEDD4L is associated with essential hypertension in female Kazakh general population: a case-control study. BMC Med Genet.

[62] Arif M, Sadayappan S, Becker RC, Martin LJ, Urbina EM (2019). Epigenetic modification: a regulatory mechanism in essential hypertension. Hypertens Res.

[63] Graham L, Padmanabhan S (2014). NEDD4L in essential hypertension. J Hypertens.

[64] Wang H-M, Li N-F, Hong J, Yao X-G, Luo W-L, Chang J-H (2010). [Association between rs4149601 polymorphism and essential hypertension in Kazakh]. Zhonghua Xin Xue Guan Bing Za Zhi.

[65] Loffing-Cueni D, Flores SY, Sauter D, Daidié D, Siegrist N, Meneton P (2006). Dietary sodium intake regulates the ubiquitin-protein ligase nedd4-2 in the renal collecting system. J Am Soc Nephrol.

[66] Wen H, Lin R, Jiao Y, Wang F, Wang S, Lu D (2008). Two polymorphisms in NEDD4L gene and essential hypertension in Chinese Hans - a population-based case-control study. Clin Exp Hypertens.

[67] Kawarazaki W, Fujita T (2021). Kidney and epigenetic mechanisms of salt-sensitive hypertension. Nat Rev Nephrol.

[68] Xu B-E, Stippec S, Chu P-Y, Lazrak A, Li X-J, Lee B-H (2005). WNK1 activates SGK1 to regulate the epithelial sodium channel. Proc Natl Acad Sci USA.

[69] Bhalla V, Daidié D, Li H, Pao AC, LaGrange LP, Wang J (2005). Serum- and glucocorticoid-regulated kinase 1 regulates ubiquitin ligase neural precursor cell-expressed, developmentally down-regulated protein 4-2 by inducing interaction with 14-3-3. Mol Endocrinol.

[70] Luzardo L, Noboa O, Boggia J (2015). Mechanisms of salt-sensitive hypertension. Curr Hypertens Rev.

[71] Ronzaud C, Loffing-Cueni D, Hausel P, Debonneville A, Malsure SR, Fowler-Jaeger N (2013). Renal tubular NEDD4-2 deficiency causes NCC-mediated salt-dependent hypertension. J Clin Invest.

[72] Aoi W, Niisato N, Sawabe Y, Miyazaki H, Marunaka Y (2006). Aldosterone-induced abnormal regulation of ENaC and SGK1 in Dahl salt-sensitive rat. Biochem Biophys Res Commun.

[73] Lifton RP, Gharavi AG, Geller DS (2001). Molecular mechanisms of human hypertension. Cell.

[74] Ishigami T, Kino T, Minegishi S, Araki N, Umemura M, Ushio H et al. Regulators of epithelial sodium channels in aldosterone-sensitive distal nephrons (ASDN): critical roles of Nedd4L/Nedd4-2 and salt-sensitive hypertension. Int J Mol Sci. 2020;21, 10.3390/ijms21113871. 10.3390/ijms21113871 PMC7312533 32485919

[75] Rizzo F, Staub O (2015). NEDD4-2 and salt-sensitive hypertension. Curr Opin Nephrol Hypertens.

[76] Kino T, Ishigami T, Murata T, Doi H, Nakashima-Sasaki R, Chen L, et al. Eplerenone-resistant salt-sensitive hypertension in Nedd4-2 C2 KO mice. Int J Mol Sci. 2017;18, 10.3390/ijms18061250. 10.3390/ijms18061250 PMC5486073 28604611

[77] Dahlberg J, Sjögren M, Hedblad B, Engström G, Melander O (2014). Genetic variation in NEDD4L, an epithelial sodium channel regulator, is associated with cardiovascular disease and cardiovascular death. J Hypertens.

[78] Snyder PM, Steines JC, Olson. DR (2004). Relative contribution of Nedd4 and Nedd4-2 to ENaC regulation in epithelia determined by RNA interference. J Biol Chem.

[79] Enslow BT, Stockand JD, Berman JM (2019). Liddle’s syndrome mechanisms, diagnosis and management. Integr Blood Press Control.

[80] Hansson JH, Nelson-Williams C, Suzuki H, Schild L, Shimkets R, Lu Y (1995). Hypertension caused by a truncated epithelial sodium channel gamma subunit: genetic heterogeneity of Liddle syndrome. Nat Genet.

[81] Rotin D, Schild L (2008). ENaC and its regulatory proteins as drug targets for blood pressure control. Curr Drug Targets.

[82] Knight KK, Olson DR, Zhou R, Snyder PM (2006). Liddle’s syndrome mutations increase Na^+^ transport through dual effects on epithelial Na^+^ channel surface expression and proteolytic cleavage. Proc Natl Acad Sci USA.

[83] Gleason CE, Frindt G, Cheng C-J, Ng M, Kidwai A, Rashmi P (2015). mTORC2 regulates renal tubule sodium uptake by promoting ENaC activity. J Clin Invest.

[84] Staub O, Dho S, Henry P, Correa J, Ishikawa T, McGlade J (1996). WW domains of Nedd4 bind to the proline-rich PY motifs in the epithelial Na^+^ channel deleted in Liddle’s syndrome. EMBO J.

[85] Goulet CC, Volk KA, Adams CM, Prince LS, Stokes JB, Snyder PM (1998). Inhibition of the epithelial Na^+^ channel by interaction of Nedd4 with a PY motif deleted in Liddle’s syndrome. J Biol Chem.

[86] Abriel H, Loffing J, Rebhun JF, Pratt JH, Schild L, Horisberger JD (1999). Defective regulation of the epithelial Na^+^ channel by Nedd4 in Liddle’s syndrome. J Clin Invest.

[87] Rotin. D (2008). Role of the UPS in Liddle syndrome. BMC Biochem.

[88] Nakamura M, Sadoshima J (2018). Mechanisms of physiological and pathological cardiac hypertrophy. Nat Rev Cardiol.

[89] Ren Z, Yu P, Li D, Li Z, Liao Y, Wang Y (2020). Single-cell reconstruction of progression trajectory reveals intervention principles in pathological cardiac hypertrophy. Circulation.

[90] Zhao D, Zhong G, Li J, Pan J, Zhao Y, Song H (2021). Targeting E3 ubiquitin ligase WWP1 prevents cardiac hypertrophy through destabilizing DVL2 via inhibition of K27-linked ubiquitination. Circulation.

[91] Bui AL, Horwich TB, Fonarow GC (2011). Epidemiology and risk profile of heart failure. Nat Rev Cardiol.

[92] Sahle BW, Owen AJ, Chin KL, Reid CM (2017). Risk prediction models for incident heart failure: a systematic review of methodology and model performance. J Card Fail.

[93] Greene SJ, Fonarow GC, Butler J (2020). Risk profiles in heart failure: baseline, residual, worsening, and advanced heart failure risk. Circulation Heart Fail.

[94] Mishra S, Kass DA (2021). Cellular and molecular pathobiology of heart failure with preserved ejection fraction. Nat Rev Cardiol.

[95] Patel Y & Joseph J. Sodium intake and heart failure. Int J Mol Sci. 2020;21, 10.3390/ijms21249474. 10.3390/ijms21249474 PMC7763082 33322108

[96] Li W, Yin L, Shen C, Hu K, Ge J, Sun A (2018). Variants: association with cardiac disorders. Front Physiol.

[97] Ge J, Sun A, Paajanen V, Wang S, Su C, Yang Z (2008). Molecular and clinical characterization of a novel SCN5A mutation associated with atrioventricular block and dilated cardiomyopathy. Circ Arrhythm Electrophysiol.

[98] Catterall. WA (2014). Sodium channels, inherited epilepsy, and antiepileptic drugs. Annu Rev Pharm Toxicol.

[99] Shen C, Xu L, Han S, Dong Z, Zhao X, Wang S (2017). Novel idiopathic DCM-related variants localised in DI-S4 predispose electrical disorders by reducing peak sodium current density. J Med Genet.

[100] McNair WP, Ku L, Taylor MRG, Fain PR, Dao D, Wolfel E (2004). SCN5A mutation associated with dilated cardiomyopathy, conduction disorder, and arrhythmia. Circulation.

[101] Shen C, Xu L, Yang Z, Zou Y, Hu K, Fan Z (2013). A1180V of cardiac sodium channel gene (SCN5A): is it a risk factor for dilated cardiomyopathy or just a common variant in Han Chinese?. Dis Markers.

[102] David Strain W, Paldánius PM (2018). Diabetes, cardiovascular disease and the microcirculation. Cardiovasc Diabetol.

[103] Cole JB, Florez JC (2020). Genetics of diabetes mellitus and diabetes complications. Nat Rev Nephrol.

[104] Cosentino F, Grant PJ, Aboyans V, Bailey CJ, Ceriello A, Delgado V (2020). 2019 ESC Guidelines on diabetes, pre-diabetes, and cardiovascular diseases developed in collaboration with the EASD. Eur Heart J.

[105] Han T, Lan L, Qu R, Xu Q, Jiang R, Na L (2017). Temporal relationship between hyperuricemia and insulin resistance and its impact on future risk of hypertension. Hypertension.

[106] Jia G, DeMarco VG, Sowers JR (2016). Insulin resistance and hyperinsulinaemia in diabetic cardiomyopathy. Nat Rev Endocrinol.

[107] Dillmann WH. Dillmann. Diabetic Cardiomyopathy. Circulation Res. 2019;124:1160–2. 10.1161/CIRCRESAHA.118.314665. 10.1161/CIRCRESAHA.118.314665 PMC6578576 30973809

[108] Shi Y, Yan C, Li Y, Zhang Y, Zhang G, Li M (2020). Expression signature of miRNAs and the potential role of miR-195-5p in high-glucose-treated rat cardiomyocytes. J Biochem Mol Toxicol.

[109] Vogel B, Claessen BE, Arnold SV, Chan D, Cohen DJ, Giannitsis E (2019). ST-segment elevation myocardial infarction. Nat Rev Dis Prim.

[110] Cahill TJ, Choudhury RP, Riley PR (2017). Heart regeneration and repair after myocardial infarction: translational opportunities for novel therapeutics. Nat Rev Drug Discov.

[111] van den Borne SWM, Diez J, Blankesteijn WM, Verjans J, Hofstra L, Narula J (2010). Myocardial remodeling after infarction: the role of myofibroblasts. Nat Rev Cardiol.

[112] Kino T, Khan M, Mohsin S. The regulatory role of t cell responses in cardiac remodeling following myocardial infarction. Int J Mol Sci 2020;21, 10.3390/ijms21145013. 10.3390/ijms21145013 PMC7404395 32708585

[113] Minegishi S, Ishigami T, Kawamura H, Kino T, Chen L, Nakashima-Sasaki R, et al. An isoform of Nedd4-2 plays a pivotal role in electrophysiological cardiac abnormalities. Int J Mol Sci. 2017;18, 10.3390/ijms18061268. 10.3390/ijms18061268 PMC5486090 28613240

[114] David JJ, Subramanian SV, Zhang A, Willis WL, Kelm RJ, Leier CV (2012). Y-box binding protein-1 implicated in translational control of fetal myocardial gene expression after cardiac transplant. Exp Biol Med (Maywood).

[115] Huang S, Li X, Zheng H, Si X, Li B, Wei G (2019). Loss of super-enhancer-regulated circRNA Nfix induces cardiac regeneration after myocardial infarction in adult mice. Circulation.

[116] Luo L, Ning F, Du Y, Song B, Yang D, Salvage SC (2017). Calcium-dependent Nedd4-2 upregulation mediates degradation of the cardiac sodium channel Nav1.5: implications for heart failure. Acta Physiol (Oxf).

[117] Shang LL, Pfahnl AE, Sanyal S, Jiao Z, Allen J, Banach K (2007). Human heart failure is associated with abnormal C-terminal splicing variants in the cardiac sodium channel. Circulation Res.

[118] Wang Y, Pan W, Bai X, Wang X, Wang Y, Yin Y (2021). microRNA-454-mediated NEDD4-2/TrkA/cAMP axis in heart failure: mechanisms and cardioprotective implications. J Cell Mol Med.

[119] Postema PG, Van den Berg M, Van Tintelen JP, Van den Heuvel F, Grundeken M, Hofman N (2009). Founder mutations in the Netherlands: SCN5a 1795insD, the first described arrhythmia overlap syndrome and one of the largest and best characterised families worldwide. Neth Heart J.

[120] Wang Q, Shen J, Splawski I, Atkinson D, Li Z, Robinson JL (1995). SCN5A mutations associated with an inherited cardiac arrhythmia, long QT syndrome. Cell.

[121] Rook MB, Evers MM, Vos MA, Bierhuizen MFA (2012). Biology of cardiac sodium channel Nav1.5 expression. Cardiovascular Res.

[122] Wilde AAM, Bezzina CR (2005). Genetics of cardiac arrhythmias. Heart.

[123] Shy D, Gillet L, Abriel. H (2013). Cardiac sodium channel NaV1.5 distribution in myocytes via interacting proteins: the multiple pool model. Biochim Biophys Acta.

[124] Benson DW, Wang DW, Dyment M, Knilans TK, Fish FA, Strieper MJ (2003). Congenital sick sinus syndrome caused by recessive mutations in the cardiac sodium channel gene (SCN5A). J Clin Invest.

[125] Abe K, Machida T, Sumitomo N, Yamamoto H, Ohkubo K, Watanabe I (2014). Sodium channelopathy underlying familial sick sinus syndrome with early onset and predominantly male characteristics. Circ Arrhythm Electrophysiol.

[126] Sergio Richter LDB, Hindricks G (2019). Atrial fibrillation ablation in heart failure. Eur Heart J.

[127] Darbar D, Kannankeril PJ, Donahue BS, Kucera G, Stubblefield T, Haines JL (2008). Cardiac sodium channel (SCN5A) variants associated with atrial fibrillation. Circulation.

[128] Lubitz SA, Yin X, Fontes JD, Magnani JW, Rienstra M, Pai M (2010). Association between familial atrial fibrillation and risk of new-onset atrial fibrillation. JAMA.

[129] Groenewegen WA, Firouzi M, Bezzina CR, Vliex S, van Langen IM, Sandkuijl L (2003). A cardiac sodium channel mutation cosegregates with a rare connexin40 genotype in familial atrial standstill. Circulation Res.

[130] Priori SG, Wilde AA, Horie M, Cho Y, Behr ER, Berul C (2013). HRS/EHRA/APHRS expert consensus statement on the diagnosis and management of patients with inherited primary arrhythmia syndromes: document endorsed by HRS, EHRA, and APHRS in May 2013 and by ACCF, AHA, PACES, and AEPC in June 2013. Heart Rhythm.

[131] Glazer AM, Wada Y, Li B, Muhammad A, Kalash OR, O’Neill MJ (2020). High-throughput reclassification of SCN5A variants. Am J Hum Genet.

[132] Chen Q, Kirsch GE, Zhang D, Brugada R, Brugada J, Brugada P (1998). Genetic basis and molecular mechanism for idiopathic ventricular fibrillation. Nature.

[133] Clatot J, Ziyadeh-Isleem A, Maugenre S, Denjoy I, Liu H, Dilanian G (2012). Dominant-negative effect of SCN5A N-terminal mutations through the interaction of Na(v)1.5 α-subunits. Cardiovascular Res.

[134] Kapplinger JD, Tester DJ, Alders M, Benito B, Berthet M, Brugada J (2010). An international compendium of mutations in the SCN5A-encoded cardiac sodium channel in patients referred for Brugada syndrome genetic testing. Heart Rhythm.

[135] Wang Y, Du Y, Luo L, Hu P, Yang G, Li T (2020). Alterations of Nedd4-2-binding capacity in PY-motif of Na 1.5 channel underlie long QT syndrome and Brugada syndrome. Acta Physiol (Oxf).

[136] Murphy LL, Moon-Grady AJ, Cuneo BF, Wakai RT, Yu S, Kunic JD (2012). Developmentally regulated SCN5A splice variant potentiates dysfunction of a novel mutation associated with severe fetal arrhythmia. Heart Rhythm.

